# Stability simulation analysis of targeted puncture in L4/5 intervertebral space for PELD surgery

**DOI:** 10.3389/fbioe.2023.1298914

**Published:** 2024-01-08

**Authors:** Yuhuai Liu, Qiongchi Zhang, Ning Ji, Jie Wang, Jie Li, Jinpei Du, Jinghao Zhao, Pengrong Ouyang, Jie Qin, Haopeng Li, Dong Wang

**Affiliations:** Department of Orthopedics, The Second Affiliated Hospital of Xi’an Jiaotong University, Xi’an, Shaanxi, China

**Keywords:** lumbar disc herniation, percutaneous endoscopic lumbar discectomy, finite element analysis, lumbar spine stability, laminotomy and facetectomy

## Abstract

**Introduction:** The application prospects of percutaneous endoscopic lumbar discectomy (PELD) as a minimally invasive spinal surgery method in the treatment of lumbar disc herniation are extensive. This study aims to find the optimal entry angle for the trephine at the L4/5 intervertebral space, which causes less lumbar damage and has greater postoperative stability. To achieve this, we conduct a three-dimensional simulated analysis of the degree of damage caused by targeted puncture-based trephine osteotomy on the lumbar spine.

**Methods:** We gathered clinical CT data from patients to construct a lumbar model. This model was used to simulate and analyze the variations in trephine osteotomy volume resulting from targeted punctures at the L4/5 interspace. Furthermore, according to these variations in osteotomy volume, we created Finite Element Analysis (FEA) models specifically for the trephine osteotomy procedure. We then applied mechanical loads to conduct range of motion and von Mises stress analyses on the lumbar motion unit.

**Results:** In percutaneous endoscopic interlaminar discectomy, the smallest osteotomy volume occurred with a 20° entry angle, close to the base of the spinous process. The volume increased at 30° and reached its largest at 40°. In percutaneous transforaminal endoscopic discectomy, the largest osteotomy volume was observed with a 50° entry angle, passing through the facet joints, with smaller volumes at 60° and the smallest at 70°. In FEA, M6 exhibited the most notable biomechanical decline, particularly during posterior extension and right rotation. M2 and M3 showed significant differences primarily in rotation, whereas the differences between M3 and M4 were most evident in posterior extension and right rotation. M5 displayed their highest stress levels primarily in posterior extension, with significant variations observed in right rotation alongside M4.

**Conclusion:** The appropriate selection of entry sites can reduce lumbar damage and increase stability. We suggest employing targeted punctures at a 30° angle for PEID and at a 60° angle for PTED at the L4/5 intervertebral space. Additionally, reducing the degree of facet joint damage is crucial to enhance postoperative stability in lumbar vertebral motion units.

## 1 Introduction

Percutaneous endoscopic lumbar discectomy (PELD) has garnered widespread recognition as an effective intervention for lumbar disc herniation (LDH), with its safety and efficacy substantiated by pertinent literature (Yeung, 1999; Takahashi et al., 2008; Liu et al., 2019; Wu et al., 2020; Ahn, 2021). Notably, potential harm to the nerve root or dural sac can be promptly discerned through intraoperative imaging and immediate patient feedback, enabling the surgeon to avert potential catastrophic complications (Zhu et al., 2017; Jia et al., 2018; Xu et al., 2019).

The different clinical approaches in nerve root decompression categorize PELD into percutaneous endoscopic interlaminar discectomy (PEID) and percutaneous transforaminal endoscopic discectomy (PTED). Additionally, there are combined surgical techniques involving both methods. The puncture and positioning procedures frequently rely on anatomical landmarks to measure partial-opening distances. A guiding needle is inserted, and intraoperative C-arm fluoroscopy is used for verification. Following precise positioning, endoscopic osteotomy with a trephine is performed to achieve decompression, thereby exposing the surgical site, which includes laminotomy and facetectomy (Wu et al., 2020; Jiang et al., 2021; Zhang et al., 2021). However, during the operation, issues related to trephine displacement and inaccurate positioning may arise. After osteotomy, the affected area may not be adequately exposed, necessitating continuous in-surgery adjustments and repeated osteotomies. This not only prolongs the surgical duration but also increases damage to bone structure and ligaments, thereby exacerbating the instability of the vertebral motion unit post-surgery and raising the likelihood of postoperative recurrence in patients.

Drawing from the anatomical characteristics of the vertebral body, the extent of osteotomy is correlated with the angle, diameter, and frequency of trephine entry. It is crucial to strike a balance between thorough lesion removal and minimizing disruption to the vertebral motion unit. Opting for less osteotomy is preferable, as it may mitigate the risk of biomechanical deterioration, and consequently, the likelihood of experiencing failed back surgery syndrome (Li et al., 2019b). In the clinical application of PELD, the careful selection of the optimal puncture angle is of paramount importance for achieving surgical minimally invasiveness, standardization, and precision (Ahn, 2019).

In light of these principles and empirical observations, we sought to analysis the variations in osteotomy volume and lumbar stability under targeted puncture-based entry angel. To determine the most suitable osteotomy entry angle with the least damage, we created a three-dimensional surgical model of the L4/5 interspace to simulate a trephine osteotomy attempt. We selected the optimal angle based on osteotomy volume and compared stress distribution and lumbar stability using a finite element analysis (FEA) model. This approach will provide valuable assistance for clinical surgical procedures.

## 2 Materials and methods

### 2.1 Simulation analysis of trephine osteotomy volume

#### 2.1.1 Lumbar model construction

This study involved 25 clinical patients. Among these participants, there were 13 males and 12 females, aged between 17 and 71 years, with an average age of 45.64 ± 12.88 years.

Inclusion criteria for participation were a clinical diagnosis of L4/5 disc herniation and receipt of PELD surgery between January 2020 and October 2021. Exclusion criteria encompassed the presence of spinal tumors, spinal fractures, infectious diseases like spinal tuberculosis, indications of multi-segmental protrusion on imaging, spinal deformities, and a history of prior spinal surgery.

The selected cases underwent preoperative thin-layer CT scanning of the L3-L5 lumbar vertebrae and intervertebral discs using a GE Lightspeed VCT 64-slice spiral CT machine from the United States, with a layer thickness of 0.625 mm. Subsequently, the DICOM (Digital Imaging and Communication of Medicine) images of the lumbar vertebrae were acquired and saved onto a CD for storage purposes. Following this, the CT images of all 25 cases were imported into Mimics 21.0 (Materialise, Inc., Leuven, Belgium). Within this platform, the three-dimensional reconstruction of the L3-L5 vertebral bodies was performed. Consequently, the resulting three-dimensional model structure was further imported into 3-matic Research 13.0 (Materialise, Inc., Leuven, Belgium) to proceed with the construction of the model.

#### 2.1.2 Trephine model construction

**FIGURE 1:**
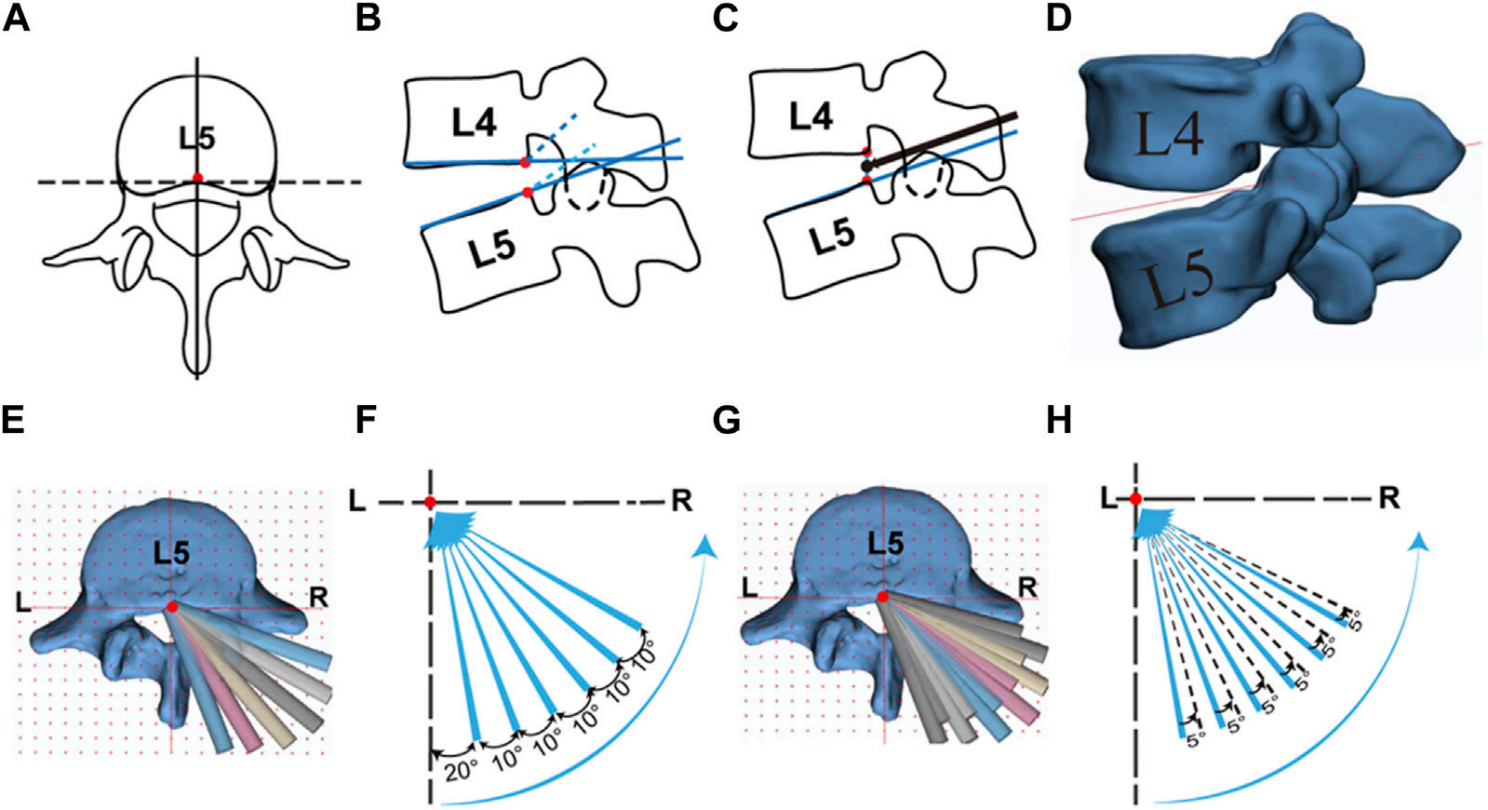
Targeted puncture approach and trephine model construction. **(A)** Midpoint of the posterior edge of L5 superior surface; **(B)** Midpoint of the inferior surface of L4 and superior surface of L5; **(C)** Trephine direction in sagittal plane; **(D)** Trephine plan in sagittal plane; **(E) (F)** Trephine osteotomy model with one attempt; **(G) (H)** Trephine osteotomy model with two attempts.

The simulation of PELD was conducted using information derived from both published literature and clinical expertise (Gadjradj et al., 2016; Kim et al., 2018; Ono et al., 2022). The lumbar models imported into 3-matic Research were constructed using the “Sketch” and “Fit plane” functions. Specifically, planes representing the inferior and superior endplates of the L4 and L5 vertebrae were established. Additionally, the midpoint of the posterior edge of the vertebral body for each endplate was determined (Figure 1A). The midpoint between the posterior edge of the inferior endplate of L4 and the posterior edge of the superior endplate of L5 was designated as the center point of the L4/5 intervertebral space (Figure 1B). A plane parallel to the superior endplate of L5 was established using this center point as reference (Figures 1C,D, Supplementary Figure S1).

In 3-matic Research, the right-sided approach was selected, with the center point of the L4/5 intervertebral space as the designated fixed point., six lines were drawn on the intervertebral reference plane, offset from the central axis by 20°/30°/40°/50°/60°/70° respectively. Based on the anatomy of the facet joint, the PEID group included 20°/30°/40°, while the PTED group included 50°/60°/70°. Cylinders with diameters of 7/8/9 mm were created to represent the clinically used trephines of the same diameters. The path taken by a single cylinder represented one osteotomy attempt of trephine (Figure 1E). On the basis of the first attempt of osteotomy, each cylinder was horizontally offset outward by 5° to simulate the second osteotomy attempt of trephine performed in clinical surgery (Figure 1F).

#### 2.1.3 Trephine osteotomy attempt

In the 3-matic Research software, the “Local Boolean” function was employed to separately calculate and record the values of the “Volume” of osteotomy during 1/2 attempts in various directions for different diameters of trephine.

### 2.2 Biomechanical finite element analysis

#### 2.2.1 Surgical model construction

**FIGURE 2:**
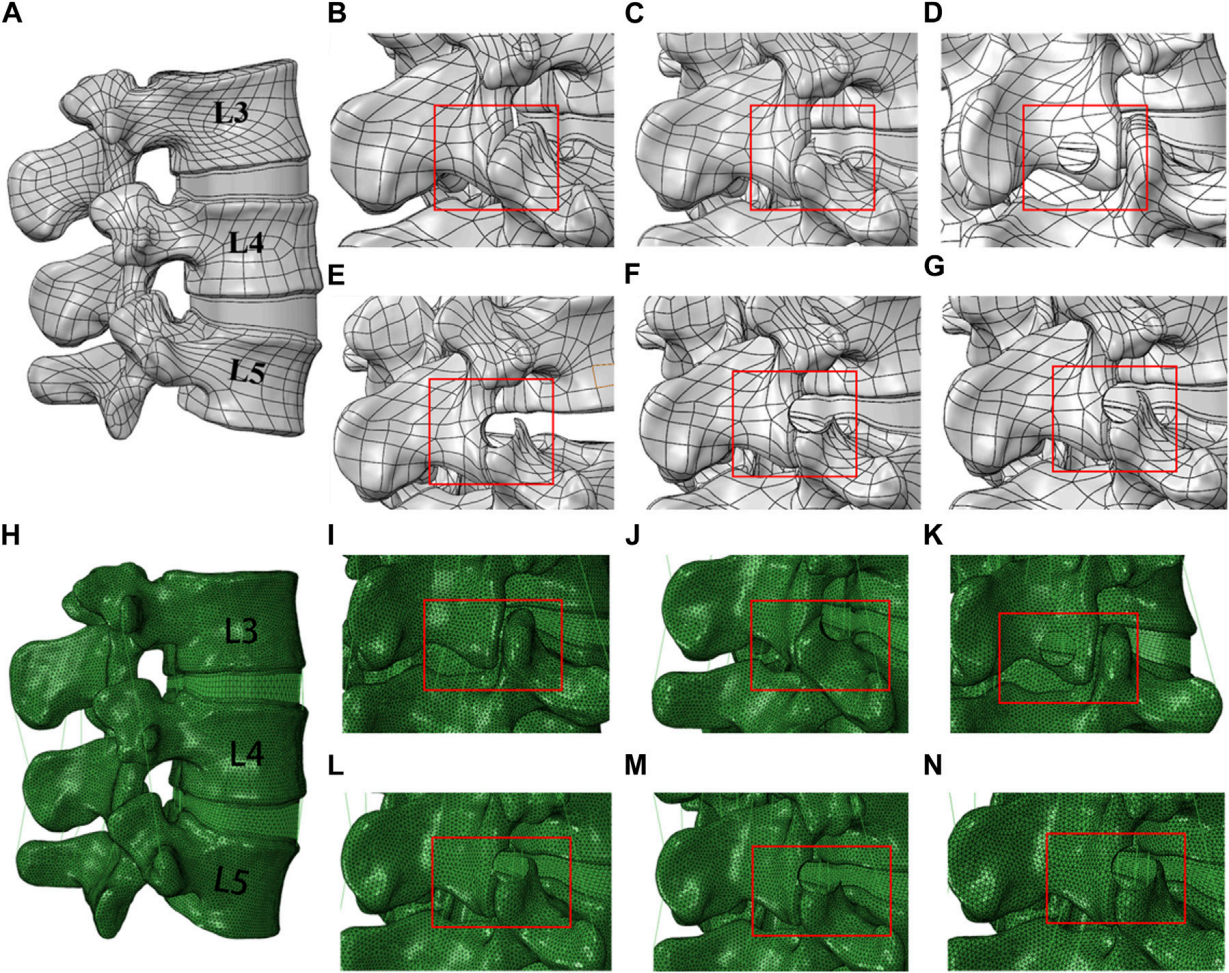
Different trephine osteotomy attempt models of the L4/5 interspace and finite element models. **(A)** Model in SOLIDWORKS; **(B)** Model 1, complete model; **(C)** Model 2, 8 mm diameter trephine, 60° osteotomy with one attempt; **(D)** Model 3, 8 mm diameter trephine, 30° osteotomy with one attempt; **(E)** Model 4, 8 mm diameter trephine, 50° osteotomy with one attempt; **(F)** Model 5, 8 mm diameter trephine, 50° osteotomy with two attempts; **(G)** Model 6, 9 mm diameter trephine, 50° osteotomy with one attempt **(H)** Model in ABAQUS; **(I–N)**: Finite element model.

We recruited one healthy male volunteer, 26 years old, with a height of 174 cm and a weight of 75 kg. Firstly, CT images were imported into Mimics to extract bone tissue, and then further optimized in 3-matic. The reconstructed model from 3-matic Research was imported into Geomagic Wrap 2017 (3D Systems, Inc. Geomagic, United States) for smoothing. Following this, cancellous bone, cortical bone, intervertebral discs, cartilage endplates, and facet joints were reconstructed using Solidworks 2020 (Dassault Systèmes, United States). We constructed a complete model of L3-L5 (Figures 2A,B).

Based on the aforementioned changes in osteotomy volume and clinical experience, we proceeded with the construction of the trephine osteotomy surgical model in Solidworks 2020. To investigate the effects of trephine angle, diameter, and number of osteotomy attempts on lumbar spine mobility and stability, we selected one attempt of osteotomy at 30° and 60° directions with an 8 mm diameter, two attempts of osteotomy at 50° direction with an 8 mm diameter, and one attempt of osteotomy at 50° direction with a 9 mm diameter. We constructed complete models and surgical models with five different trephine osteotomy attempts for subsequent analysis (Figures 2C–G).

#### 2.2.2 Finite element model construction

The surgical models were imported into HyperMesh 14.0 (Altair Technologies, Inc. Carlsbad, CA, United States). The lumbar finite element model was developed through the addition of ligaments, mesh separation, and the assignment of respective material properties, setting loading conditions, and performing finite element analysis in Abaqus 2018 (Dassault Systèmes, United States). The material properties for various lumbar structures were obtained from literature sources (Li et al., 2019a; Li et al., 2019b; Li et al., 2020; Qin et al., 2022) (Table 1; Figure 2H-N). M1 is a complete model with intact ligaments and cartilage. The other models are achieved through the corresponding removal of ligaments and facet joint cartilage. Using a trephine osteotomy involves removing a specific area of facet joint cartilage. At the facet joint connections, four ligaments are set. In M2, a quarter of these ligaments are removed, while in M4 and M5, half are removed. M5 has a larger area of cartilage removed than M4. M6 involves removing three-quarters of the ligaments, along with extensive cartilage removal. M3 is achieved by removing the right ligamentum flavum.

**TABLE 1:** The finite element material properties.

	Young’s modulus (MPa)	Poisson’s ratio	Cross sectional area (mm2)
Cortical bone	12,000	0.3	
Cancellous bone	100	0.2	
endplate	23.8	0.4	
cartilage	10	0.4	
Annulus fibrosus matrix	4.2	0.45	
Nucleus pulposus	0.4	0.499	
Annulus fibrosus fiber	455	0.3	1.35
Anterior longitudinal ligament	7.8	0.3	63.7
Posterior longitudinal ligament	10	0.3	20
Yellow ligament	15	0.3	40
Interspinous ligament	10	0.3	40
supraspinous ligament	8	0.3	30
ligamenta intertransversaria	10	0.3	1.8
ligamenta capsulare	7.5	0.3	30

The cortical bone exhibited an approximate thickness of 1 mm. The combined cross-sectional area of the annulus fibrosus, nucleus pulposus, and endplates occupied approximately 95% of the corresponding vertebral cross-sectional area. Specifically, the cross-sectional area of the nucleus pulposus represented 40% of the intervertebral disc. In order to ascertain the relative position of the nucleus pulposus, a ratio of 1.62 was established by comparing the distance from the front edge of the annulus fibrosus to the nucleus pulposus with the distance from the rear edge of the annulus fibrosus to the nucleus pulposus. Furthermore, the thickness of the facet joint cartilage measured less than 1 mm (Zhao et al., 2018; Li et al., 2020; Liu et al., 2023). The element types for cortical bone, cancellous bone, endplates, facet joint cartilage, and annulus fibrosus were C3D4, while the element type for the nucleus pulposus was C3D4H, and for ligaments, it was T3D2 (Supplementary Figure S3). Table 2 presents the node and element counts for the three models.

**TABLE 2:** Number of nodes and elements in the models.

Model	Nodes	Elements
M1	290,951	853,687
M2	291,084	854,557
M3	291,185	854,016
M4	290,714	852,621
M5	290,077	851,375
M6	290,132	851,631

#### 2.2.3 Boundary conditions, loading methods

The inferior surface of the L5 vertebra was constrained, and a follower load of 400N was applied to the superior surface of the L3 vertebra to simulate physiological compression loading. Additionally, a torque of 10Nm was applied to L3 to simulate six types of lumbar activities including flexion, extension, lateral flexion, and rotation (Fan et al., 2019; Wang et al., 2020; Qin et al., 2022). The lumbar spine mobility and maximum von Mises stress on the L4/L5 intervertebral space were calculated through finite element analysis.

The statistical results of the experimental data were analyzed using SPSS version 26.0. The experimental data were expressed as mean ± standard deviation. Adobe Illustrator 2021 (Adobe Inc. United States) and PowerPoint were used for data visualization. *t*-test analysis was employed for comparing results between two sample sets, and ANNOVA analysis was used for multiple sample analysis.

## 3 Results

### 3.1 Variation in osteotomy volume

**FIGURE 3:**
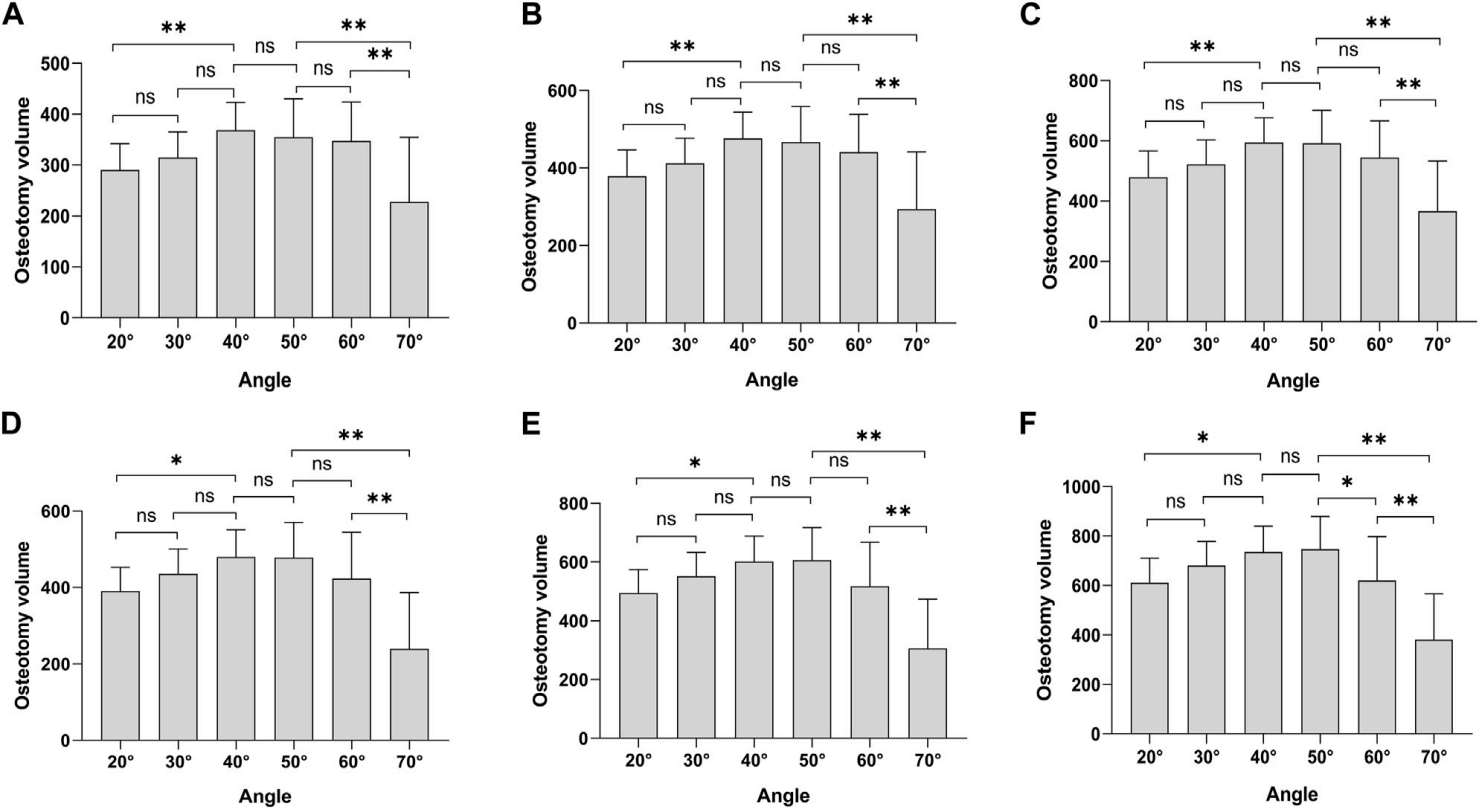
Osteotomy volume. **(A)** 7 mm diameter, one attempt; **(B)** 8 mm diameter, one attempt; **(C)** 9 mm diameter, one attempt; **(D)** 7 mm diameter, two attempts; **(E)** 8 mm diameter, two attempts; **(F)** 9 mm diameter, two attempts. *p < 0.05, **p < 0.01.

We conducted a statistical analysis of trephine osteotomy volume (Figure 3). Within the PEID group, the largest osteotomy volume was observed at a 40° angle, compared to the smallest volume at a 20° angle. Furthermore, no statistically significant difference in osteotomy volume was found between 20° and 30° angles (*p* > 0.05). However, a significant difference was observed between the 20° and 40° angles (*p* < 0.05), regardless of the number of trephine attempts. Across all groups, the osteotomy volume did not significantly differ between 30° and 40° angles (*p* > 0.05).

Within the PTED group, the largest osteotomy volume was observed at a 50° angle, with the smallest occurring at a 70° angle. Except for cases with a 9 mm diameter involving two osteotomy attempts, a statistically significant difference in osteotomy volume was noted between the 50° and 60° angles (*p* < 0.05) (Figure 3F). In contrast, no significant differences were found in other groups (*p* > 0.05). Additionally, there was a significant difference in osteotomy volume between the 70° angle and both the 50° and 60° angles (*p* < 0.05).

Moreover, when comparing the PEID and PTED groups, we observed that for two trephine attempts with an 8/9 mm diameter, the osteotomy volume at a 40° angle was smaller than that at a 50° angle (Figures 3E,F). In all other cases, the osteotomy volume at 40° exceeded that at 50°, and there was no statistically significant difference in osteotomy volume between these two angles (*p* > 0.05).

### 3.2 Model validity verification

**FIGURE 4:**
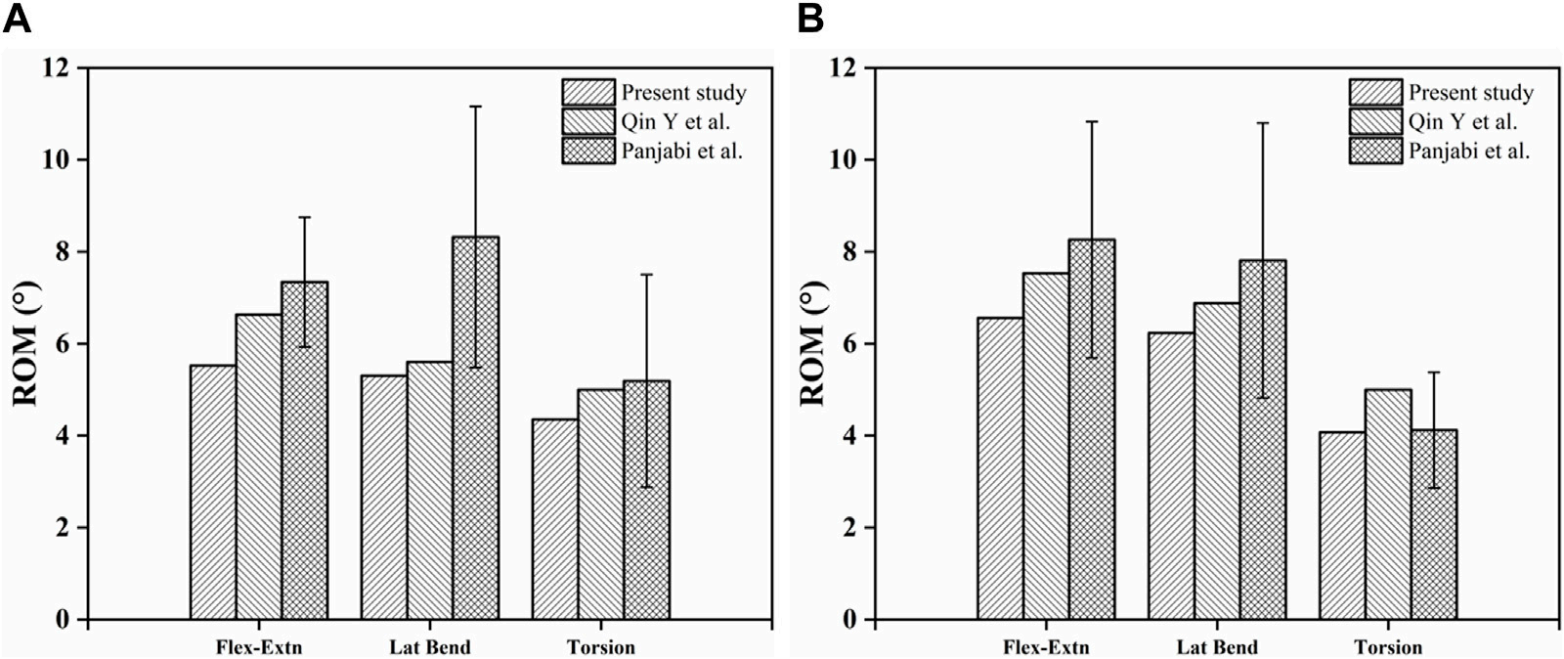
Comparison of ROM in finite element models. **(A)** L3-L4 ROM in Model 1; **(B)** L4-L5 ROM in Model 1.

We compared the Range of Motions at the L3-L4 and L4-L5 levels with the findings from a previous cadaveric study and finite element analysis (Fan et al., 2019; Wang et al., 2020). Our results closely corroborated theirs. Consequently, the normal, intact lumbar FE model in this study can be employed for subsequent analyses (Figure 4).

### 3.3 Measurement of L4-L5 range of motion

**FIGURE 5:**
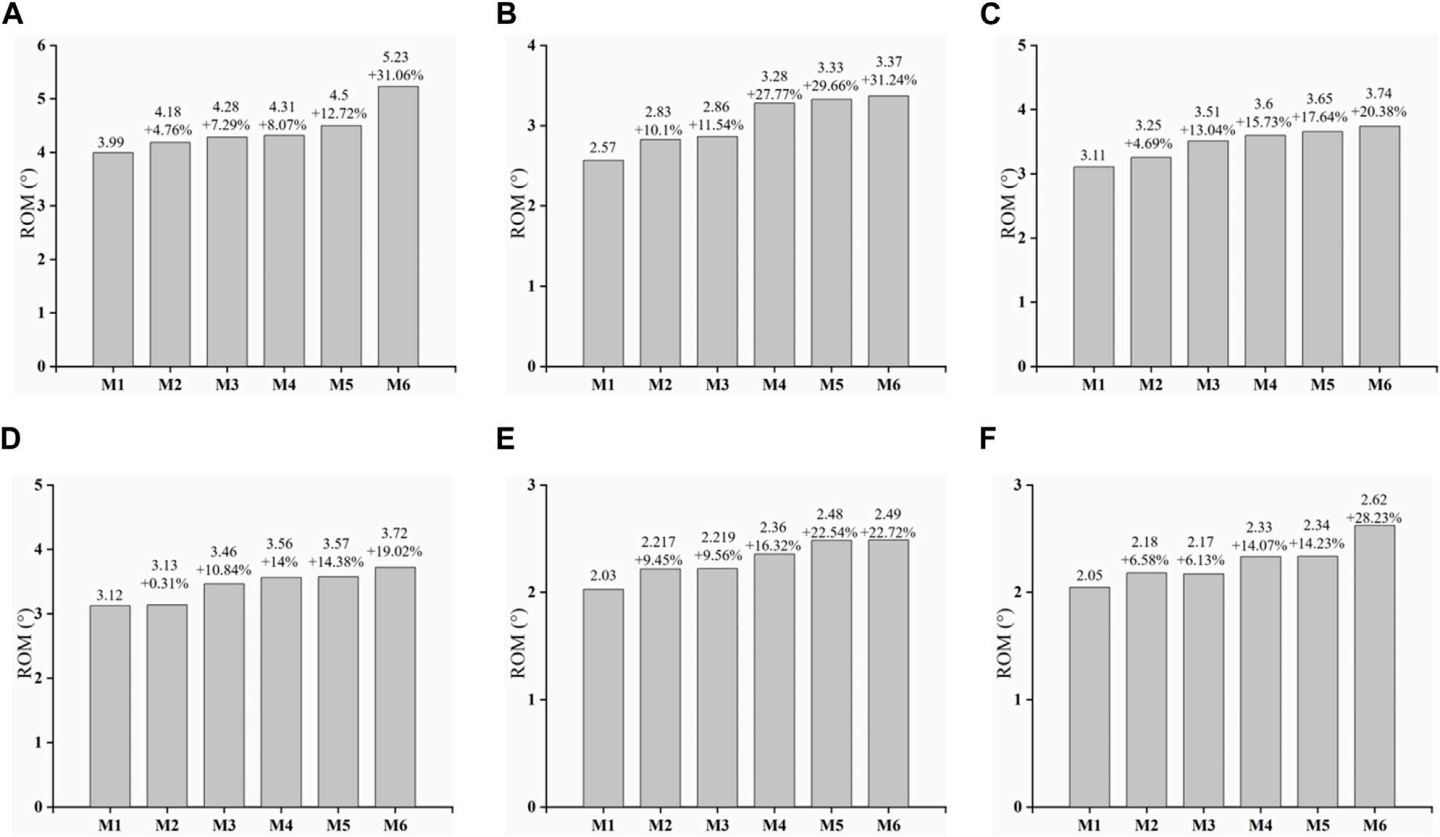
Variation of L4-L5 ROM. **(A)** Flexion **(B)** extension **(C)** left bending **(D)** right bending **(E)** left rotation **(F)** right rotation.

Compared to the Model 1, there is an increasing trend in the L4-L5 range of motion across each model. Model 6 demonstrates the most substantial change in activity, with significant percentage increases in both anteflexion and posterior extension. Model 2 and Model 3 exhibit noticeable differences in left and right bending, while changes in other directions are less significant. Models 4 and 5 show a marked increase in activity specifically in the left rotation direction, with smaller differences in other directions (Figure 5).

### 3.4 Stress analysis of L4/5 annulus fibrosus and endplate

**FIGURE 6:**
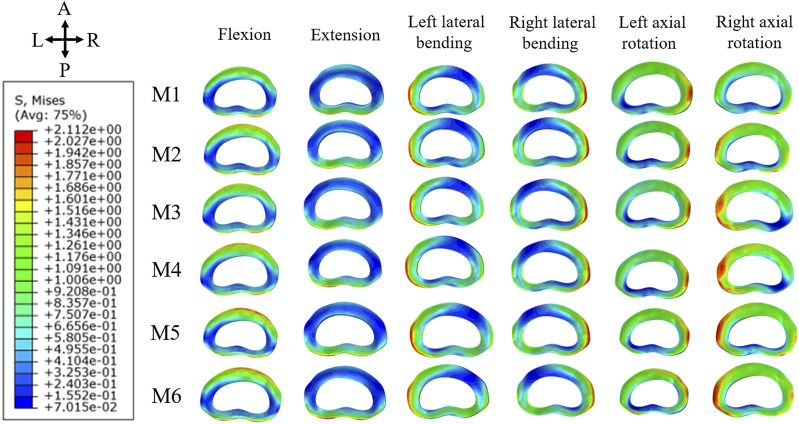
Maximum von Mises stress distribution map of L4/5 annulus fibrosus.

In the analysis of maximum von Mises stress within the L4/5 annulus fibrosus, M6 consistently showed the highest stress levels across all six directions. Notably, there was a significant increase in stress during anteflexion, posterior extension, and right rotation. In contrast, M2 and M3 demonstrated the most significant stress differences in posterior extension and left rotation, with smaller discrepancies in other directions. M4 and M5 exhibited their highest stress levels primarily in posterior extension, with more subtle variations in other directions. Figure 6 illustrates the stress distribution map of the L4/5 annulus fibrosus, highlighting areas of maximum von Mises stress concentration.

**FIGURE 7:**
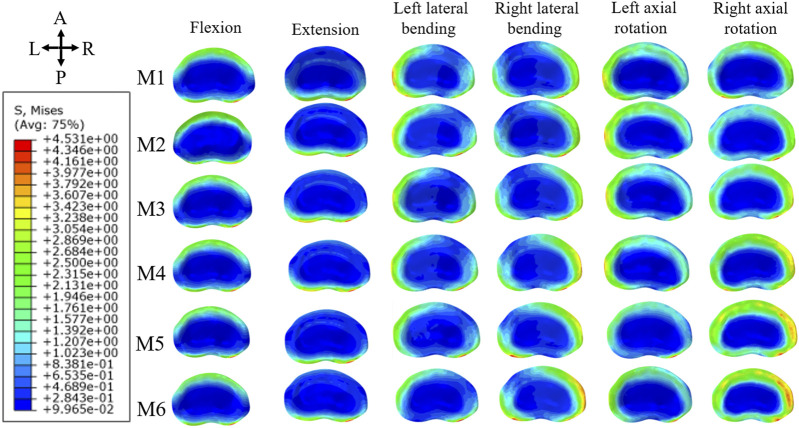
Maximum von Mises stress distribution map of superior endplate of L5.

In terms of maximum von Mises stress on the L5 superior endplate, M6 exhibited the highest values across all six directions, with particularly notable stresses during extension, left rotation, and right rotation. The stress differences in extension between M2 and M3 were minimal, whereas they were more pronounced in other directions. For M4 and M5, significant variations were observed in right rotation, with less marked differences in other directions. Figure 7 displays the stress distribution map of the L5 superior endplate, showing areas of maximum von Mises stress concentration.

**FIGURE 8:**
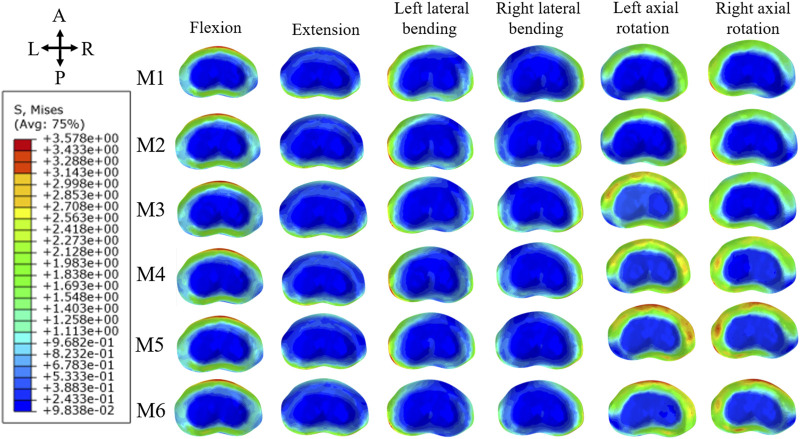
Maximum von Mises stress distribution map of inferior endplate of L4.

Regarding the maximum von Mises stress on the L4 inferior endplate, the overall increase in stress was less significant compared to that on the L5 superior endplate. Consistent with previous findings, M6 displayed the highest stress values in all six directions, with extension showing the greatest increase. For M2 and M3, the increase in stress was particularly noticeable in right rotation, while it was less pronounced in other directions. For M4 and M5, the increase in stress was relatively minor across all directions. Figure 8 illustrates the stress distribution map of the L4 inferior endplate, indicating areas of maximum von Mises stress concentration.

The overall assessment of stability in this study was centered on the L4/5 intervertebral space. We computed various parameters, including ROM and maximum von Mises stress on the annulus and endplates, to evaluate the effectiveness of different surgical models. The inferior endplates of L4 exhibited minimal variation in von Mises stress in contrast to the superior endplate of L5. Additionally, significant changes were observed in the ROM of the lumbar vertebrae. The most pronounced biomechanical deterioration was seen in M6, especially in extension and right rotation. M2 and M3 showed noticeable differences, mainly in rotation. The disparities in M3 and M4 were primarily evident in extension and right rotation. Similar patterns were noted in the stress analysis of adjacent segments (Supplementary Tables S1–S3). M6 displayed marked instability, predominantly in flexion, extension, and right rotation. M2 and M3 varied in stress distribution at the L4 superior endplate, with M2 experiencing a lesser increase in maximum von Mises stress compared to M3. M4 and M5 showed similar degrees of stress increase.

## 4 Discussion

The use of trephine osteotomy is crucial in PELD procedures. Previous studies have highlighted the importance of meticulous osteotomy for significant postoperative symptom relief (Kotheeranurak et al., 2023). However, considering its minimally invasive nature, minimizing damage to the spinal motion unit and reducing the risk of postoperative recurrence is essential. Furthermore, developing precise angle designs for PELD surgeries in clinical practice continues to be a challenge. To address this, we have quantified the extent of damage based on the vertebral body’s anatomical characteristics and the principles of minimally invasive treatment. This quantification enables a visual representation of stability changes, providing vital guidance for clinical surgeons in both their surgical techniques and the management of postoperative patient recovery.

In this experimental design, the puncture approach is directed towards the L4/5 intervertebral space, making it suitable for the majority of clinical herniation types. According to the classification of lumbar disc herniation zones (Mysliwiec et al., 2010), the PEID targeted approach is suitable for types 1-A, 1-B, 2-A, and 2-B, but may not be as effective for special types such as extreme lateral and bilateral protrusions (Lee and Lee, 2008; Pan et al., 2016). The PTED targeted approach is applicable for types 1-C, 2-B, 2-AB, 2-C, and 3-AB, but may not be ideal for cases involving enlarged transverse processes of L5, high iliac crests, narrow intervertebral foramen, or various complex types of massive or highly displaced disc herniations (Pan et al., 2016; Depauw et al., 2018).

This study found that in the L4/5 interspace, the osteotomy volume was the largest in the 50° direction compared to other directions. This result is attributed to the necessity of passing through the facet joints in this direction. Relevant research indicates that damaging the facet joints significantly increases spinal instability. As the diameter of the trephine increases, the extent of osteotomy also expands, leading to a corresponding decrease in stability. This not only significantly affects local degeneration but also impacts the mobility of adjacent vertebrae (Waguespack et al., 2002; Adams and Roughley, 2006; Muto, 2011; Li et al., 2019b; Li et al., 2021).

In the PEID group, the osteotomy volume was smaller in the 20° direction compared to the 40° direction. However, the 20° direction posed a challenge of excessive inward deviation, implying that the trephine would come close to the base of the spinous process, increasing the surgical difficulty (Supplementary Figure S2). Additionally, while the 20° direction may serve as an option for interlaminar access, there is a risk of damaging the dura mater and causing cerebrospinal fluid leakage, with limitations on the surgical field. In contrast, the 40° direction had a larger osteotomy volume and was closer to the facet joints. However, using a large-diameter trephine or performing multiple osteotomy attempts may increase the risk of damaging the facet joints. Therefore, we recommend using the 30° direction for access, as its osteotomy volume does not significantly differ from the 20° direction. This approach aligns with the surgical habits of clinical practitioners and provides a certain buffer space.

In the PTED group, the main difference lies in the extent of damage to the facet joints. For instance, in the 70° direction, due to its proximity to the lateral aspect of the intervertebral foramen, the osteotomy volume is relatively small. However, this may not be sufficient to meet the decompression needs, similar to the YESS technique (Yeung, 1999), making its indications relatively limited. As for the 60° direction, the angle primarily targets the superior facet joint of the L5 vertebra for osteotomy. Although an increasing diameter may cause some degree of damage to the inferior facet joint of L4, the overall volume of the osteotomy is still less than that in the 50° direction. Therefore, we recommend choosing the 60° direction.

The FEA method, used for simulating biomechanical changes in lumbar vertebrae, is known for its repeatability, low cost, and simplicity. Consequently, it has gained widespread use in biomechanical studies (Liu et al., 2023; Nikkhoo et al., 2023). Changes in vertebral mobility and the maximum von Mises stresses have a close relationship with stability (Khalaf and Nikkhoo, 2021; Nikkhoo et al., 2021). In this experiment, M2 induced damage to the facet joint capsule, and M3 induced damage to the ligamentum flavum, respectively. This resulted in a noticeable increase in extension mobility compared to M1. While M3 showed greater changes in mobility compared to M2, the difference was minimal. Consequently, it cannot be conclusively determined whether the 60° direction is superior to the 30° direction in a single attempt, indicating the need for further studies with a larger sample size. For M4 and M5, a significant increase in mobility was observed in the extension and left rotation directions, although the differences between them in these directions were minor. In horizontal movements, the number of osteotomy attempts appeared to have minimal impact on mobility. However, performing two attempts as opposed to a single attempt at 50° resulted in a more extensive range of osteotomy, effectively removing residual parts of the L5 superior facet joint. This clinical approach is beneficial in preventing the compression of exiting nerve roots caused by any remaining bone in the area. M2 retained a portion of the L5 superior facet joint and a part of the attached ligamentous capsule, thereby rendering it more stable than M4. This retention also aids in preventing postoperative re-compression. In this study, M6 demonstrated the lowest stability. Compared to M4, M6 with larger trephine diameter resulted in the removal of a greater amount of facet cartilage and ligaments, significantly decreasing facet joint stability (Wangsawatwong et al., 2023).

By calculating the maximum von Mises stresses in the annulus fibrosus of the L4/5 intervertebral disc, and in the inferior endplate of L4 as well as the superior endplate of L5, we can analyze the risk of surgical recurrence. Areas of stress concentration suggest that repeated strain could accelerate damage in these regions, potentially serving as primary factors in surgical recurrence. Similar to the changes in mobility, the maximum von Mises stresses in various models show a significant increase in extension and rotational directions. In model M6, the annulus fibrosus undergoes abnormally high stress levels. This indicates severe damage to the facet joint capsule, accompanied by considerable ligament and articular cartilage removal, resulting in increased compressive deformation of the vertebrae and cartilage.

Observing the distribution map of maximum von Mises stresses, we note that stress concentrates at the anterior and posterior edges of the superior endplate of L5 in flexion. The tension at the posterior edge is more pronounced than the pressure at the anterior edge, a trend that mirrors the stress distribution in the annulus fibrosus of the L4/5 intervertebral space. In contrast, during extension, the anterior edge experiences significantly less tension. Endplates play a pivotal role in distributing pressure. Stress concentration heightens the risk of micro-fractures in the lower part of the endplate and impairs nutrient diffusion between the endplates, which is essential for the metabolism of adult intervertebral discs. Accelerated disc degeneration consequently increases the risk of natural degeneration (Ruberté et al., 2009; Chepurin et al., 2022; Wang et al., 2022; Zhou et al., 2022).

However, the study has several limitations worth noting. Firstly, the use of CT data from healthy adult males for our FEA models may not be universally applicable, especially for patients with several pathological changes, such as osteoporosis and multiple-segment degeneration (Chuang et al., 2012; Chien et al., 2014). These conditions involve different densities and mechanical properties in both cortical and cancellous bones (Al-Barghouthi et al., 2020; Garay et al., 2022). Without distinguishing between these bone structures, stress analysis and volume calculations might be prone to inaccuracies, a factor that is also dependent on the analytical algorithms of analysis software. Secondly, modeling the intervertebral disc as merely an elastic material with a fiber-reinforced annulus fibrosus is a simplification. Degenerated discs exhibit altered mechanical properties and geometrical characteristics, including reduced water content, changed collagen structure, and decreased height, which affect load distribution and spinal stability (Volz et al., 2022). As studied by Elmasry et al., the intervertebral disc displays poroelastic behavior due to its fluid-saturated nature (Elmasry et al., 2017; Elmasry et al., 2018). Poroelastic models offer a more accurate representation of the disc’s response to mechanical loads, especially regarding fluid flow, pressure distribution, and long-term biomechanical behavior. Relying on a simplistic model could lead to inaccuracies in depicting the biomechanical response of the intervertebral disc, particularly under cyclic loading or prolonged stress. Thirdly, soft tissues such as muscles play an important role in maintaining the biomechanical balance of the skeleton, especially in sustaining spinal stability and affecting lumbar load (El Bojairami and Driscoll, 2022). We concur that incorporating detailed musculoskeletal structures, especially back muscles like the multifidus, would substantially enhance the accuracy and predictive capabilities of our spine models. The role of these structures in contributing to the stability and biomechanics of the lumbar spine is indeed crucial. Fourthly, the study by Amirouche et al. indeed offers valuable insights pertinent to our work (Amirouche et al., 2015). We concur that testing on human specimens provides results that closely resemble real-life scenarios. Utilizing FEA visualization in conjunction with cadaveric specimen validation can yield highly convincing results. However, due to our experiments’ extensive design, using cadaveric specimens would lead to significantly higher workload and costs. Fifthly, we can make a preliminary judgment on the trephine angle from the frontal and sagittal positions, but the precise angle derived from simulation analysis requires a comprehensive comparison of the relationship between trephine angle and human body surface positioning in clinical application. While our experimental validation is not exhaustive, our innovative approach to design still provides substantial assistance to clinical surgical practices. Despite the limitations in employing cadaveric specimens for validation, we believe that our work contributes meaningful insights to the field.

In our study, we calculated the osteotomy volume and observed general trends of lumbar stability through FEA. This simplified approach was based on balancing the need for detailed, patient-specific modeling with the practical constraints of computational resources and the current state of the art in finite element analysis. Our method facilitates trend-based analysis, illustrating the impact of trephine attempt angle on stability and aiding in predicting postoperative degeneration in patients.

## 5 Conclusion

Through simulation analysis of the PELD surgical procedures, it is advisable to opt for a 30° direction in PEID and a 60° direction for trephine osteotomy in PTED at the L4/5 intervertebral space to minimize lumbar spine damage. Moreover, to achieve better lumbar stability and reduce the risk of postoperative recurrence, it is recommended to limit the number of trephine osteotomy attempts and minimize damage to the lumbar facet joints.

## Data Availability

The original contributions presented in the study are included in the article/Supplementary Material, further inquiries can be directed to the corresponding authors.
