# Supplementary material for: Stability simulation analysis of targeted puncture in L4/5 intervertebral space for PELD surgery
Source: Front Bioeng Biotechnol. 2024 Jan 8;11:1298914. doi: 10.3389/fbioe.2023.1298914 (PMC10800398; doi:10.3389/fbioe.2023.1298914)
Supplement: Supplementary file 1 [file DataSheet1.docx]

Supplementary Material

Stability Simulation Analysis of Targeted Puncture in L4/5 Intervertebral Space for PELD Surgery

Yuhuai Liu^1†^, Qiongchi Zhang^1†^, Ning Ji^1^, Jie Wang^1^, Jie Li^1^, Jinpei Du^1^, Jinghao Zhao^1^, Pengrong Ouyang^1^, Jie Qin^1^, Haopeng Li^1^*, Dong Wang^1^*

*** Correspondence:** Dong Wang: wado110@163.com

Haopeng Li: lihaopeng3993@163.com

# Supplementary Tables

Supplementary Table 1 L3 inferior endplate maximum von Mises stress (MPa)

|  | M1 | M2 | M3 | M4 | M5 | M6 |
| --- | --- | --- | --- | --- | --- | --- |
| Flexion | 1.41 | 1.63  +15.92% | 1.82  +29% | 2.03  +43.99% | 2.09  +48.47% | 2.77  +96.66% |
| Extension | 4.03 | 4.07  +0.84% | 4.37  +8.25% | 4.60 +13.98% | 5.02  +24.34% | 5.87  +45.54% |
| Left lateral | 2.73 | 2.76  +0.99% | 2.94  +7.65% | 2.98  +9.01% | 3.09  +13.26% | 3.13  +14.57% |
| Right lateral | 2.91 | 3.02  +3.53% | 3.05  +4.67% | 3.27  +12.05% | 3.28  +12.63% | 3.33  +14.34% |
| Left axial | 1.45 | 1.47  +1.10% | 1.62  +11.56% | 1.75  +20.17% | 1.88  +29.53% | 2.00  +37.58% |
| Right axial | 1.20 | 1.41  +17.38% | 1.52  +27.15% | 1.63  +36.26% | 1.64  +36.93% | 1.72  +43.36% |

Supplementary Table 2 L4 superior endplate maximum von Mises stress (MPa)

|  | M1 | M2 | M3 | M4 | M5 | M6 |
| --- | --- | --- | --- | --- | --- | --- |
| Flexion | 2.11 | 2.36  +11.77% | 2.49  +18.32% | 2.55  +20.98% | 2.74  +29.90% | 2.99  +42.10% |
| Extension | 2.25 | 2.53  +12.31% | 2.78  +23.41% | 2.82  +25.23% | 2.97  +32.07% | 3.42  +51.93% |
| Left lateral | 1.82 | 1.85  +1.59% | 2.15  +18.42% | 2.16  +18.86% | 2.18  +19.90% | 2.24  +23.31% |
| Right lateral | 1.66 | 1.68  +1.27% | 1.91  +15.27% | 1.92  +15.99% | 2.01  +21.48% | 2.01  +21.54% |
| Left axial | 1.09 | 1.10  +0.74% | 1.12  +2.58% | 1.12  +3.40% | 1.30  +19.14% | 1.45  +33.76% |
| Right axial | 0.88 | 0.89  +1.45% | 1.14  +29.63% | 1.22  +38.87% | 1.37  +55.85% | 1.41  +61.21% |

Supplementary Table 3 L3/4 annulus fibrosus maximum von Mises stress (MPa)

|  | M1 | M2 | M3 | M4 | M5 | M6 |
| --- | --- | --- | --- | --- | --- | --- |
| Flexion | 0.93 | 1.12  +21.14% | 1.17  +26.32% | 1.18  +27.73% | 1.41  +52.56% | 1.67  +80.74% |
| Extension | 1.47 | 1.62  +10.57% | 1.72  +17.11% | 1.83  +24.81% | 1.84  +25.43% | 2.11  +43.97% |
| Left lateral | 0.95 | 1.01  +5.92% | 1.09  +14.32% | 1.10  +15.26% | 1.14  +19.46% | 1.14  +19.57% |
| Right lateral | 0.89 | 0.90  +2.08% | 0.96  +8.26% | 1.02  +15.35% | 1.03  +16.15% | 1.05  +19.08% |
| Left axial | 0.75 | 0.76  +0.58% | 0.79  +4.94% | 0.83  +10.18% | 0.85  +12.23% | 0.86  +13.97% |
| Right axial | 0.76 | 0.7617  +0.67% | 0.80  +5.59% | 0.81  +6.52% | 0.85  +12.98% | 0.86  +13.22% |

# Supplementary Figures


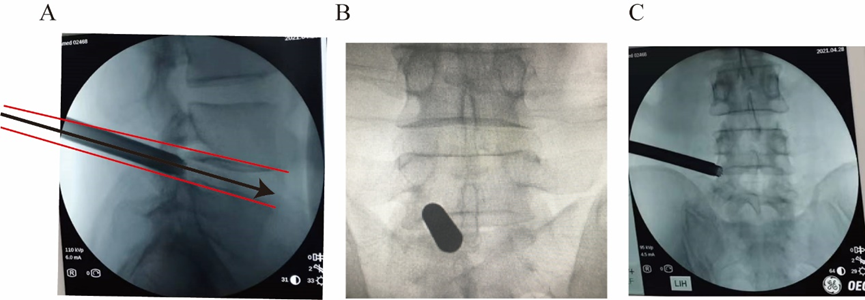


**Supplementary Figure 1.** Trephine positioning of PELD surgical. (A) sagittal view (B)PEID (C)PTED


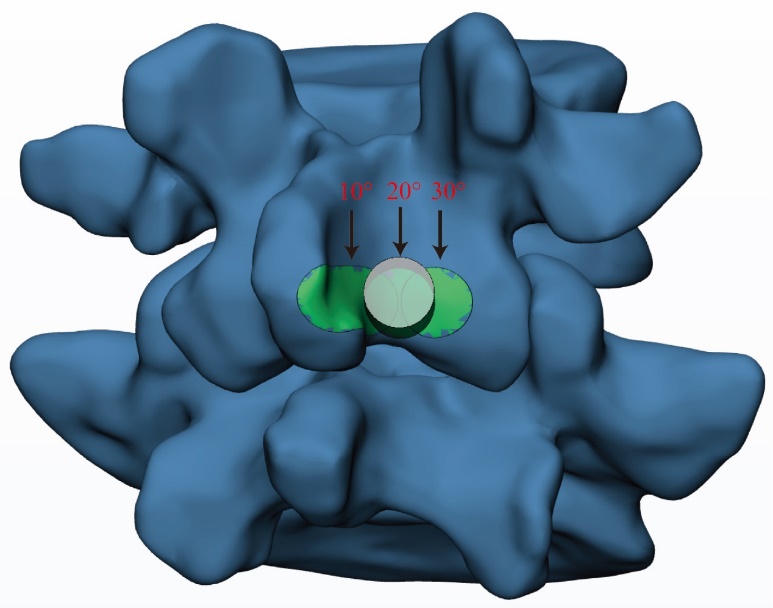


**Supplementary Figure 2.** Different trephine osteotomy angle


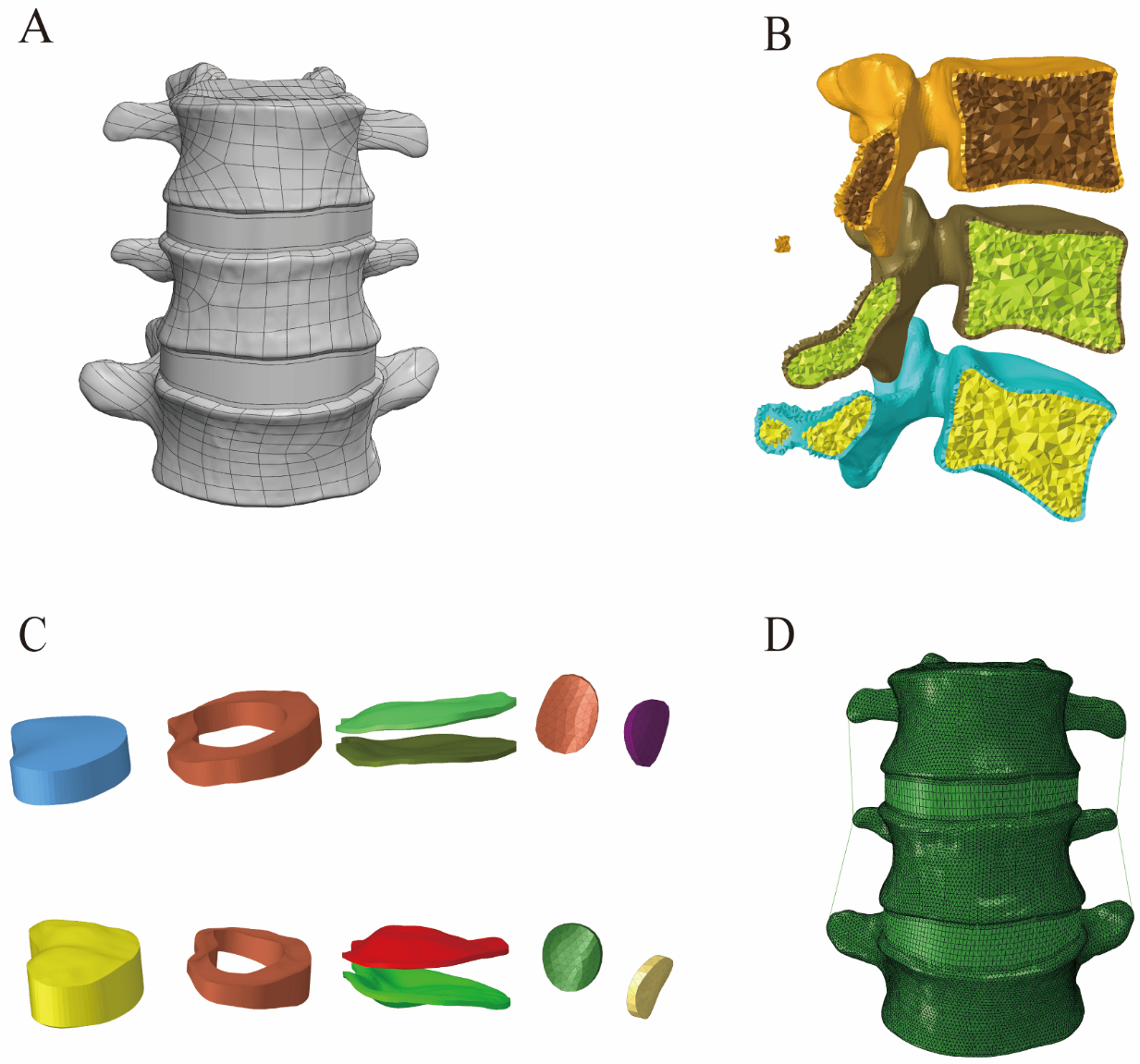


**Supplementary Figure 3.** Finite Element Analysis Model Construction. (A)Model in SOLIDWORKS (B) Cortical bone and cancellous bone (C) Nucleus pulposus, annulus fibrosus, endplate, facet joint articular cartilage (D) Intact finite element model
